# Enhanced triacylglycerol production in the diatom *Phaeodactylum tricornutum* by inactivation of a Hotdog-fold thioesterase gene using TALEN-based targeted mutagenesis

**DOI:** 10.1186/s13068-018-1309-3

**Published:** 2018-11-12

**Authors:** Xiahui Hao, Ling Luo, Juliette Jouhet, Fabrice Rébeillé, Eric Maréchal, Hanhua Hu, Yufang Pan, Xiaoming Tan, Zhuo Chen, Lingjie You, Hong Chen, Fang Wei, Yangmin Gong

**Affiliations:** 1 Key Laboratory of Biology and Genetic Improvement of Oil Crops, Ministry of Agriculture, Oil Crops Research Institute of Chinese Academy of Agricultural Sciences, No. 2 Xudong Second Road, Wuhan, 430062 People’s Republic of China; 2 Hubei Key Laboratory of Lipid Chemistry and Nutrition, Oil Crops Research Institute of Chinese Academy of Agricultural Sciences, Wuhan, 430062 China; 3 Laboratoire de Physiologie Cellulaire et Végétale, Centre National de la Recherche Scientifique, Commissariat à l’Energie Atomique et aux Energies Alternatives, Institut National de la Recherche Agronomique, Université Grenoble Alpes, UMR 5168, 38041 Grenoble, France; 4 Key Laboratory of Algal Biology, Institute of Hydrobiology, Chinese Academy of Sciences, Wuhan, 430072 China; 5 Hubei Collaborative Innovation Center for Green Transformation of Bioresources, Hubei Key Laboratory of Industrial Biotechnology, College of Life Sciences, Hubei University, Wuhan, 430062 China; 6 Shandong Provincial Key Laboratory of Plant Stress, College of Life Science, Shandong Normal University, Jinan, 250014 China

**Keywords:** Acyl-CoA thioesterase, Acyl-CoA, Fatty acids, *Phaeodactylum tricornutum*, TALEN, Triacylglycerols

## Abstract

**Background:**

In photosynthetic oleaginous microalgae, acyl-CoA molecules are used as substrates for the biosynthesis of membrane glycerolipids, triacylglycerol (TAG) and other acylated molecules. Acyl-CoA can also be directed to beta-oxidative catabolism. They can be utilized by a number of lipid metabolic enzymes including endogenous thioesterases, which catalyze their hydrolysis to release free fatty acids. Acyl-CoA availability thus plays fundamental roles in determining the quantity and composition of membrane lipids and storage lipids.

**Results:**

Here, we have engineered the model diatom *Phaeodactylum tricornutum* to produce significantly increased TAGs by disruption of the gene encoding a Hotdog-fold thioesterase involved in acyl-CoA hydrolysis (ptTES1). This plastidial thioesterase can hydrolyze both medium- and long-chain fatty acyl-CoAs, but has the highest activity toward long-chain saturated and monounsaturated fatty acyl-CoAs. The maximum rate was found with oleoyl-CoA, which is hydrolyzed at 50 nmol/min/mg protein. The stable and targeted interruption of acyl-CoA thioesterase gene was achieved using a genome editing technique, transcription activator-like effector nucleases (TALENs). Disruption of native ptTES1 gene resulted in a 1.7-fold increase in TAG content when algal strains were grown in nitrogen-replete media for 8 days, whereas the content of other lipid classes, including phosphoglycerolipids and galactoglycerolipids, remained almost unchanged. The engineered algal strain also exhibited a marked change in fatty acid profile, including a remarkable increase in 16:0 and 16:1 and a decrease in 20:5. Nitrogen deprivation for 72 h further increased TAG content and titer of the engineered strain, reaching 478 μg/10^9^ cells and 4.8 mg/L, respectively. Quantitative determination of in vivo acyl-CoAs showed that the total acyl-CoA pool size was significantly higher in the engineered algal strain than that in the wild type.

**Conclusions:**

This study supports the role of ptTES1 in free fatty acid homeostasis in the plastid of *Phaeodactylum* and demonstrates the potential of TALEN-based genome editing technique to generate an enhanced lipid-producing algal strain through blocking acyl-CoA catabolism.

**Electronic supplementary material:**

The online version of this article (10.1186/s13068-018-1309-3) contains supplementary material, which is available to authorized users.

## Background

Some photosynthetic microalgae synthesize and accumulate large amounts of triacylglycerols (TAGs) and deposit them into lipid droplets (LDs) under stress conditions, especially nutrient deprivation. As one of energy dense compounds, these biologically synthesized TAG molecules have gained great attention owing to the potential to be used as liquid biofuel feedstock [1, 2]. Up to now, despite its technological feasibility, microalgae-to-biofuel technology remains significantly challenging and economic viability is hampered by energetic costs and sustainability issues. One major hurdle is the low biomass and oil productivities of the selected algal strains. Engineering and optimization of algal strains and metabolic pathways provide a powerful tool and efficient strategy to increase oil productivity, with the promise of advancing the research and development of economically feasible algae-to-biofuel technology [3].

Over the past 10 years, the oleaginous diatom *Phaeodactylum tricornutum* has been used as a microalgal model to engineer lipid metabolism. Some important advances include increased accumulation of shorter chain length fatty acids by heterologous expression of plant-derived fatty acyl-ACP thioesterases [4], and enhanced accumulation of high-value omega-3 long-chain polyunsaturated fatty acids by expression of heterologous desaturase and elongase [5]. In addition, enhanced lipid production was achieved via heterologous co-expression of a yeast diacylglycerol acyltransferase and a plant oleosin [6], overexpression of malic enzyme [7], glycerol-3-phosphate acyltransferase [8, 9], targeted knockdown of phosphoenolpyruvate carboxykinase [10], disruption of the UDP-glucose pyrophosphorylase gene [11], overexpression of a gene involved in nitric oxide emission via the by-production of fumaric acid [12] and inhibition of acetyl-CoA utilization for sterol metabolism [13, 14]. These studies for enhancing lipid production in *P. tricornutum* can be classified into two different approaches: (1) overexpressing enzymes, especially acyltransferases, to increase acyl flux being channeled to TAG synthesis pathway or enhance reductant (NADPH) supply for lipogenic enzymes; (2) inhibition of or blocking off competing pathway to decrease lipid catabolism or increase substrate supply. In eukaryotic microalgae, there are two primary metabolic pathways for TAG biosynthesis: acyl-CoA-dependent pathway and acyl-CoA-independent pathway. In the first pathway, which is also known as the Kennedy pathway, three molecules of acyl-CoAs are used as substrates for successive acylation of glycerol backbone at *sn*-1, -2 and -3 positions by three distinct acyltransferases, leading to the formation of one molecule of TAG. The final and committed step of TAG biosynthesis is catalyzed by acyl-CoA:diacylglycerol acyltransferase (DGAT). In acyl-CoA-independent pathway, phospholipid:diacylglycerol acyltransferase (PDAT) catalyzes the transfer of a fatty acyl moiety from the *sn*-2 position of phosphatidylcholine (PC) to the *sn*-3 position of DAG to form TAG and *sn*-1 lyso-PC. DGAT is considered a key enzyme for biotechnological purpose to enhance lipid production [15]. The potential of using diatom DGATs from *Phaeodactylum* and *Nannochloropsis* for engineering of lipid content has been demonstrated in both native and heterologous hosts [16, 17]. While DGAT activity contributes greatly to acyl flux toward TAG synthesis, acyl-CoA allocation can also be influenced by other enzymes. In eukaryotic cells, acyl-CoAs can be metabolized by at least six major enzyme families: desaturases and elongases, dehydrogenases, acyl-CoA thioesterases, carnitine palmitoyltransferases and lipid acyltransferases [18]. Of these metabolic enzymes, acyl-CoA thioesterases catalyze the hydrolysis of acyl-CoA thioesters to free fatty acids and coenzyme A (CoASH). The expression of acyl-CoA thioesterases has been detected in most cellular compartments, such as cytosol, endoplasmic reticulum, mitochondria and peroxisomes. Acyl-CoA thioesterases may regulate cellular lipid metabolism by controlling the composition and concentration of acyl-CoA, CoASH and free fatty acids, although their physiological functions remain largely unknown [19]. Based on the structural features and functional implication, acyl-CoA thioesterases include the α/β-fold hydrolase and the Hotdog-fold thioesterase/dehydratase subfamilies. The Hotdog subfamily contains a structurally conserved fold consisting of a seven-stranded antiparallel β-sheet ‘bun’ that wraps around a five-turn-α-helical ‘sausage’ [20]. In this study, we identified a plastid-localized Hotdog-fold thioesterase ptTES1 in the diatom *P. tricornutum*, which exhibits in vitro acyl-CoA hydrolytic activity toward various chain length saturated and polyunsaturated acyl-CoAs ranging from C_3_ to C_20_. We further inactivated the ptTES1 gene and observed a great increase in TAG content. Despite the compromised growth, the *ptTES1* knockout mutant showed an increased TAG productivity when compared to wild-type strain. This study demonstrates the potential of TALEN-mediated knockout of target genes as an effective strategy to enhance TAG production in oleaginous diatoms.

## Results

### Acyl-CoA thioesterase activity of ptTES1

**Fig. 1:**
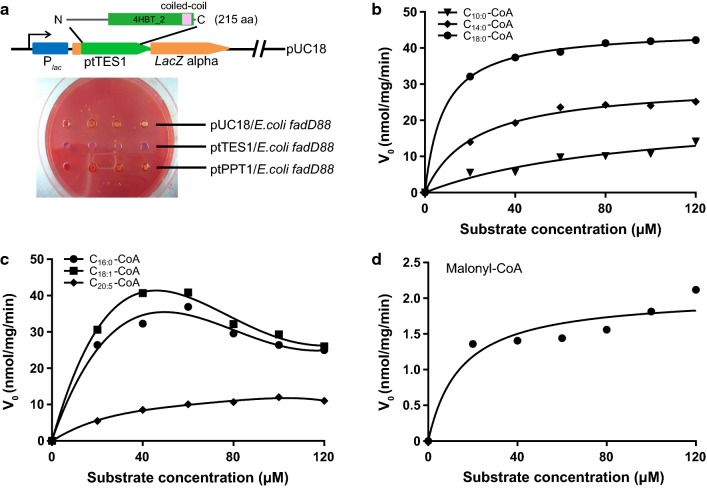
Acyl-CoA thioesterase activity of ptTES1. **a** ptTES1 was expressed in frame with *lacZ* (upper) in *E. coli* K27 *fadD* mutant fadD88 and resulted in the color change of *E. coli* colonies grown on M9 agar plate containing neutral red. Colonies that express an active ptTES1 are dark pink, while those containing empty vector pUC18 or expressing palmitoyl protein thioesterase ptPPT1 (*J10454*) are light yellow. The time-dependent hydrolysis by ptTES1 of varied concentrations of decanoyl-CoA (10:0), myristoyl-CoA (14:0), and stearoyl-CoA (18:0) (**b**); palmitoyl-CoA (16:0), oleoyl-CoA (18:1), and eicosapentaenoyl-CoA (20:5) (**c**); and malonyl-CoA (3:0) (**d**); was monitored by measuring the change of *A*_420_. Reactions were carried out at 37 °C in mixture including 10 mM Hepes (pH 7.5), 50 mM KCl, and 0.3 mM DTNB

In acyl-CoA-dependent lipid biosynthetic pathways, acyl-CoA availability is crucial for the final quantity and composition of storage lipids such as TAGs. Intracellular acyl-CoAs may be subject to desaturation, elongation, hydrolysis, and acyl transfer to various acyl acceptors, all of which contribute to acyl-CoA availability. Acyl-CoA can also be directed to mitochondria and/or peroxisomes to undergo fatty acid beta-oxidation. Hotdog family thioesterases have been reported to catalyze the hydrolysis of acyl-CoAs and these thioesterases generally show broad acyl-CoA substrate specificities. In the diatom *P. tricornutum*, there are at least four thioesterase-like proteins, of which two proteins are predicted to contain a PaaI_thioesterase domain, one belongs to the 4-HBT-II thioesterase subfamily and the last one is annotated to be a palmitoyl protein thioesterase (ptPPT). Our previous study showed that heterologous expression of the 4-HBT-II subfamily thioesterase (ptTES1) in *E. coli fad*D88 strain induced a higher level of accumulation of both stearic (C18:0) and oleic (C18:1) acids when compared to the control strain harboring the empty plasmid [21]. In this study, we further verify the thioesterase activity of ptTES1 using agar plate-based examination. We first expressed ptTES1 as *lac*Z N-terminal fusion under control of the *lac* promotor in *E. coli* mutant K27 (*fadD88*) (Fig. 1a), which lacks acyl-CoA synthetase and thus is deficient in the degradation of free fatty acids [22]. When plated on neutral red-containing agar, there was a color change in colony between *E. coli fadD88* strains expressing active thioesterases and those expressing inactive thioesterases or empty plasmid. Expression of an active thioesterase results in the release of free fatty acids, which cannot be reactivated in the *E. coli* K27 strain. It can be reasoned that accumulation of free fatty acids will lower the pH of the agar medium and thereby result in a change of colony color in the presence of the pH indicator neutral red. As shown in Fig. 1a, we observed an obvious difference in the colors of colonies expressing ptTES1 and palmitoyl protein thioesterase (ptPPT1) or empty plasmid (pUC18). This agar plate-based examination further demonstrates in vivo thioesterase activity of ptTES1 in *E. coli*.

To determine the substrate specificity of ptTES1 for acyl-CoAs, we then overexpressed codon-optimized ptTES1 fused to the 6×His tag in *E. coli* Rosetta (DE3) to perform in vitro enzyme assay. Recombinant ptTES1-His_6_ was purified by affinity chromatography. SDS/PAGE analysis provided an estimated protein mass of 25 kDa. Using purified ptTES1, we explored its capacity to hydrolyze various acyl-CoAs with different carbon chain lengths and saturations. The thioesterase activity of purified ptTES1 with acyl-CoAs as substrates was monitored spectrophotometrically based on time-dependent increase in *A*_412_ values, following the formation of 5-thio-2-nitrobenzoate by a rapid reaction between DTNB [5,5′-dithiobis-(2-nitrobenzoic acid)] and the released free CoA. Additional file 1: Figure S1 shows the activity of ptTES1 at a fixed protein concentration (4–5 μg) using various concentrations of acyl-CoAs with different chain lengths as substrates. Following mixing of ptTES1 with palmitoyl-CoA (C16:0), stearoyl-CoA (C18:0) or oleoyl-CoA (C18:1), time-dependent increase in *A*_412_ values was nearly linear; however, the rates of increase in *A*_412_ and the maximal *A*_412_ values varied according to different acyl-CoA concentrations (Additional file 1: Figure S1A–D). Although time-dependent increase in *A*_412_ values for myristoyl-CoA (C14:0) and C14:0-CoA was also observed at some substrate concentrations, absorbance values levelled off during the 80 min time course for substrate concentrations lower than 60 or 80 μM (Additional file 1: Figure S1E, F). We investigated the influence of acyl-CoA molecular species on thioesterase activity of purified ptTES1. The saturation curves for various acyl-CoAs are plotted and shown in Fig. 1b–d. As shown in Fig. 1b, the saturation curves for C10:0-, C14:0-, C18:0-CoAs demonstrate that the values of *V*_0_ for these saturated acyl-CoAs increased linearly up to substrate concentrations of 120 μM. The values of *V*_0_ for C20:5-CoA also increased in proportion to substrate concentrations and the saturation curve was very similar to that of C10:0-CoA (Fig. 1c). Substrate inhibition was observed for C16:0- and C18:1-CoAs and the values of *V*_0_ for these two acyl-CoAs increased up to concentrations of 60 μM and then declined (Fig. 1c). While the values of *V*_0_ for malonyl-CoA were one magnitude lower than that for other fatty acyl-CoAs, they increased up to a concentration of 120 μM (Fig. 1d). Kinetic constants and values of *K*_m_, *V*_max_, *k*_cat_ and *k*_cat_/*K*_m_ are summarized in Table 1. The values of *V*_max_ and *K*_m_ for acyl-CoAs longer than C10-CoA were in the range of 16.38–50.75 nmol/min/mg/protein and 8.24–91.76 μM, respectively. Of these acyl-CoAs tested in this study, stearoyl-CoA (C18:0) exhibited the lowest *K*_m_ value (8.24 μM) and the highest *k*_cat_/*K*_m_ value (2371 M^−1^ s^−1^). Purified ptTES1 showed very low thioesterase activity toward malonyl-CoA and even no activity toward acetyl-CoA, both of which are starting substrates for de novo fatty acid biosynthesis in plastid. These data indicate that although purified ptTES1 has a thioesterase activity toward a broad range of acyl-CoA thioesters, it has a strong preference for stearoyl-CoA over other long-chain saturated and monounsaturated acyl-CoAs (C16:0 and C18:1-CoAs).

**Table 1:** Kinetic constants and characteristics of ptTES1-catalyzed acyl-CoA hydrolysis

Substrate	*K*_m_ (μM)	*V*_max_ (nmol/min/mg)	*k*_cat_ (S^−1^)	*k*_cat_/*K*_m_ (M^−1^ s^−1^)
Malonyl-CoA	14.64	2.05	0.0009	60.68
Decanoyl-CoA (C10:0)	91.76	22.83	0.0099	107.81
Myristoyl-CoA (C14:0)	21.79	30.25	0.0131	601.58
Palmitoyl-CoA (C16:0)	14.76	45.32	0.0196	1330.53
Stearoyl-CoA (C18:0)	8.246	45.12	0.0196	2371.09
Oleoyl-CoA (C18:1)	12.37	50.75	0.0220	1777.82
Eicosapentaenoyl-CoA (C20:5)	38.61	16.38	0.0071	183.84

### Generation of *ptTES1* knockout mutant using TALEN-based technique

**Fig. 2:**
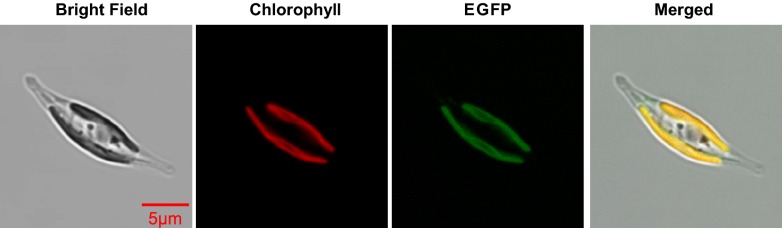
Localization of full-length ptTES1:eGFP fusion protein expressed in *P. tricornutum*. Bright field, light microscopical images; chlorophyll, chlorophyll auto-fluorescence; eGFP, GFP fluorescence; merged, merged channel. The scale bar represents 5 μm

**Fig. 3:**
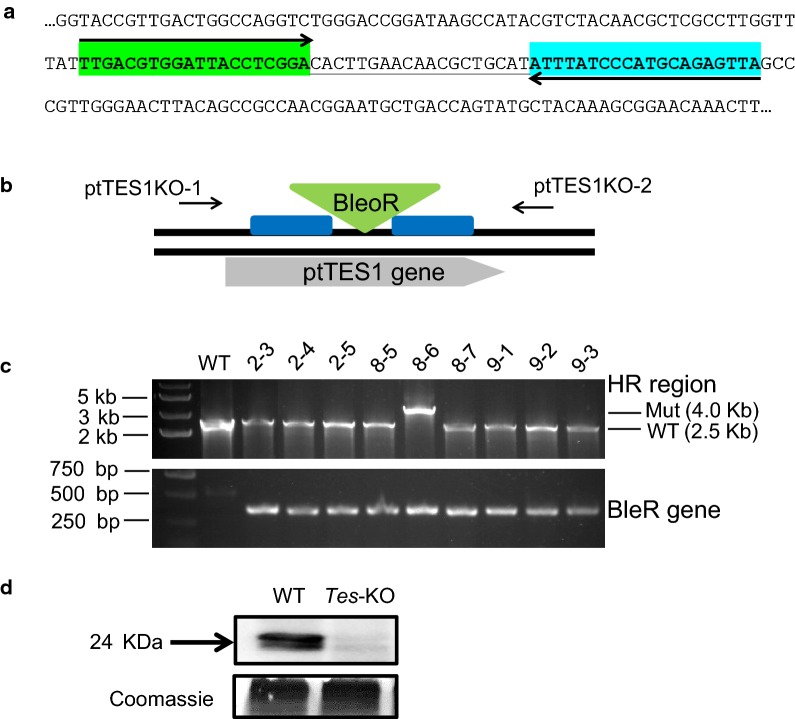
Inactivation of the ptTES1 gene using TALEN-based genome editing technique. **a** Selection of TALEN binding sequences for ptTES1 knockout. A region located in the 4HBT_2 domain of ptTES1 was selected and the 59 nt region consisted of 20 nt for the upstream TALEN (green), a 19 nt spacer where nuclease cleavage may occur, and 20 nt for the downstream TALEN (light blue). Homology regions flanking the Zeocin resistance cassette were designed outside the 59 nt binding region for homologous recombination. **b** Diagram of ptTES1 gene with targeted disruption by the *ShBle* cassette. The BleoR resistance cassette (green triangle) was flanked by appropriately 1-kb homology regions (blue boxes) on both sides, and the primer pair ptTES1KO-1/ptTES1KO-2 was used to detect the insertion of the ShBle cassette into the ptTES1 gene. **c** PCR was used to screen *P. tricornutum* colonies co-transformed with both TALEN expression plasmid and *ShBle* knockout plasmid. **d** Western blotting was used to detect the ptTES1 expression in the *ptTES1* knockout mutant and wild-type algal strain. Total soluble proteins were extracted from both strains, separated by 12% SDS-PAGE, blotted, and incubated with the α-ptTES1 antibody. Total soluble proteins were separated and stained with Coomassie brilliant blue as a loading control

To examine the subcellular localization of ptTES1, we fused the full-length amino acids to eGFP. *P. tricornutum* transformants expressing the ptTES1-eGFP construct showed GFP fluorescence mainly in the plastid (Fig. 2), where de novo fatty acid synthesis takes place. Furthermore, we performed cell fractionation and western blot with α-ptTES1 antibody. The native ptTES1 protein could be detected in the plastid as well as in the cytosol (Additional file 2: Figure S2). The GFP fluorescence detection and immunoblot result do not correlate with *in silico* predictions of subcellular localization. Indeed, there is no evidence of a bipartite transit peptide made of a signal peptide [23], followed by a chloroplast-transit peptide [24], nor of an ASAFAP motif involved in plastid targeting in *Phaeodactylum* [25]. Rather, pTES1 was predicted to be targeted to the mitochondrion, based on the Mitoprot predictive tool [26]. Although *P. tricornutum* ptTES1 was identified to have acyl-CoA thioesterase activity in vitro as purified protein, whether it is directly involved in the hydrolysis of acyl-CoAs in the plastid in vivo remains unknown. To determine whether inactivation of *ptTES1* has an effect on fatty acid and lipid synthesis in *P. tricornutum*, we constructed *ptTES1* knockout mutants using TALEN-based genome editing technology as previously described [27]. We first designed and constructed a TALEN plasmid that targeted a pair of *ptTES1* TALEN binding sequences which are spaced by a 19-nt space region (Fig. 3a). A knockout (KO) plasmid was then constructed to target the *ShBle* cassette to the putative TALEN cutting site by homologous recombination (Fig. 3b). Both TALEN and KO-plasmids were co-transformed into *P. tricornutum* cells by electroporation. We obtained one positive *ptTES1* knockout line from about 30 transformants by PCR screening (Fig. 3c), and Western blot analysis showed a complete lack of ptTES1 expression in this positive knockout line that was designated *Tes*-KO (Fig. 3d).

### Inactivation of *ptTES1* led to alteration of fatty acid profile and increase in TAG accumulation

**Fig. 4:**
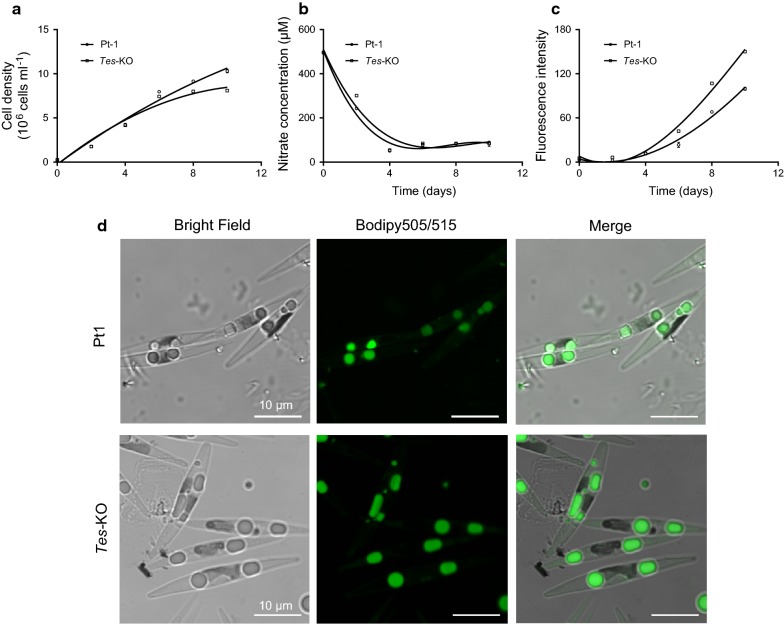
Effect of the *ptTES1* gene inactivation on cell growth and lipid accumulation in *P. tricornutum*. Cell density (**a**), nitrate concentration in culture media (**b**), and accumulation of TAGs (**c**) detected by Nile Red assay (fluorescence intensity normalized to cell number) of wild-type and the ptTES1 knockout mutant (*Tes*-KO) were analyzed at different days during the growth in F/2-enriched artificial seawater medium. Error bars represent SD of three biological replicates. **d** Microscopy images (above, wild-type Pt1; below, the *ptTES1* knockout mutant *Tes*-KO) of Bodipy 505/515-stained algal cells grown in F/2 enriched seawater medium with nitrogen deprivation for 72 h

Wild-type *P. tricornutum* strain Pt-1 and *Tes*-KO knockout line were first analyzed for growth rate and lipid accumulation in nitrogen-replete F/2 media. The *Tes*-KO knockout line exhibited slower growth than wild-type cells from day 4 to 10 (Fig. 4a). We measured nitrate concentration in the media and found that wild-type and the *Tes*-KO knockout line showed almost the same rate of nitrogen consumption. During the initial 4 days, nitrate concentration sharply decreased from 195 μM to around 50 μM, and nitrate was nearly exhausted at day 4; it maintained a relatively stable level of 83 μM during the following 6 days in both wild-type and mutant cultures (Fig. 4b). Using the Nile Red staining method, we measured fluorescence intensity, which corresponds to the amount of neutral lipids accumulated in algal cells. Neutral lipid accumulation was observed from day 4 to 10, during which the nitrate became limited; the amount of neutral lipids in *Tes*-KO knockout line was more than that in wild-type cells (Fig. 4c). To examine whether inactivation of *ptTES1* has an effect on the formation of lipid droplet (LD), we further stained algal cells grown in nitrogen-deprived F/2 media using the fluorescent staining Bodipy505/515. Upon nitrogen deprivation, mature LDs could be observed in both wild-type and mutant cells. In a typical wild-type *P. tricornutum* cell, nitrogen deprivation for 72 h resulted in the formation of two LDs with an average diameter of 1.98 μm. In contrast to wild-type cell, the number of LDs in a representative *Tes*-KO mutant cell remained unchanged but their average diameter reached 2.59 μm (Fig. 4d).

**Fig. 5:**
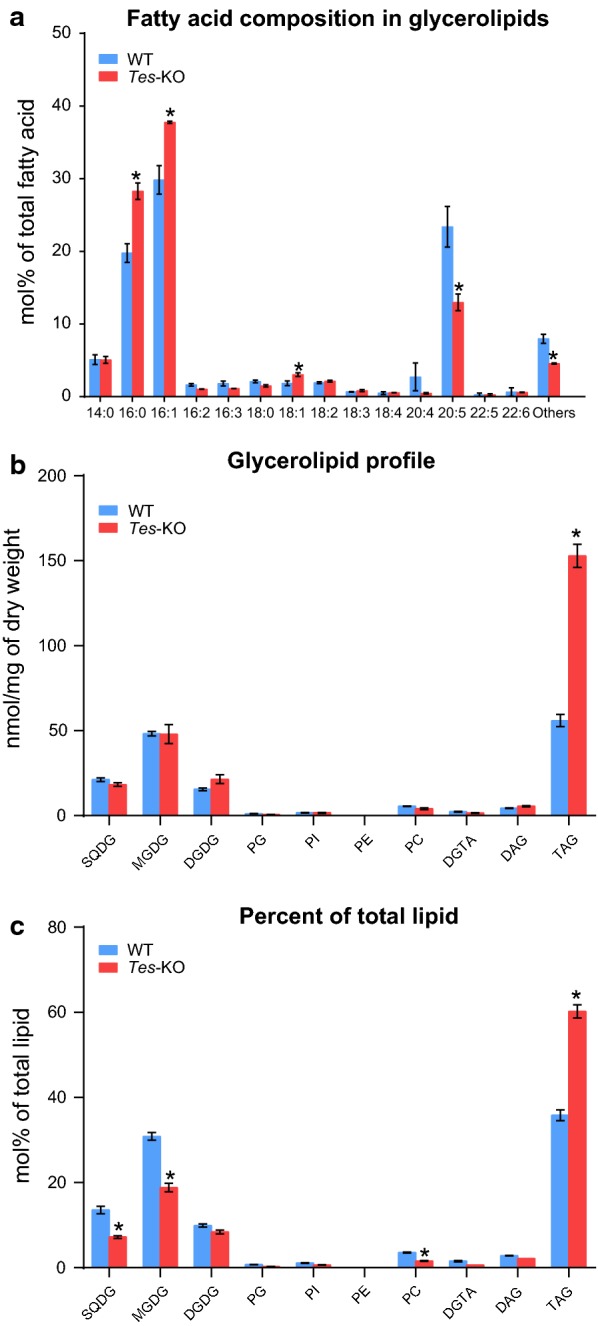
Quantitative analysis of glycerolipids from *P. tricornutum* wild type and the *ptTES1* knockout mutant. Data are the means of three biological replicates ± SD. Asterisks indicate the significant difference compared with WT (*t* test, *P < 0.05). **a** Fatty acid profile of total lipid extracts; **b** quantitative analysis of various glycerolipid classes. Glycerolipids are expressed in nmol/mg dry cell weight. **c** Glycerolipid profile in total lipid extract. Glycerolipid proportions are given in percentages

To determine whether lipid synthesis was affected by inactivation of *ptTES1*, we quantitatively analyzed glycerolipids isolated from the *ptTES1* mutant and wild-type cells grown in nitrogen-replete F/2 media for 8 days. This time point was chosen because it corresponds to the moment when the *ptTES1* mutant had similar growth rate as the wild type and oil accumulation had occurred in both strains. After glycerolipids were extracted, polar and neutral fractions were separated by TLC, and the molar proportion of each fraction was quantified by GC–FID after transmethylation to generate fatty acid methyl esters. The major fatty acids of *P. tricornutum* WT cells are C14:0, C16:0, C16:1 and C20:5, which comprise more than 70% of the total fatty acids in the stationary growth phase. C18 fatty acids including C18:0, C18:1, C18:2, C18:3 and C18:4 account for only 5% of the total fatty acids. Compared to the fatty acid composition of WT, in the *ptTES1* mutant C16:0 was increased from 20 to 29% and C16:1 was increased from 30 to 38%. Both C16:0 and C16:1 are synthesized in the stroma of *Phaeodactylum* plastid [28] where ptTES1 is located. By contrast, C20:5, which is synthesized by a series of elongases and desaturases located in the endomembranes [28], was decreased from 23 to 13% (Fig. 5a). The total fatty acid content of 222 μg/mg dry cell weight (dcw) in *Tes*-KO cells was significantly higher than 188 μg/mg dcw in WT cells. Consistent with the increase in fluorescence intensity and the diameter of LDs, the TAG content in total glycerolipids extracted from *Tes*-KO cells was increased by 1.7-fold when compared to WT (from 56 to 153 nmol/mg dcw). There was no significant difference between the contents of other lipid classes, including SQDG, MGDG, DGDG, PG, PI, PE, PC and DAG, in WT and *Tes*-KO mutant cells (Fig. 5b). For the proportion of each lipid class in total glycerolipids, TAG was increased from 35% in WT to 60% in the mutant, whereas SQDG, MGDG and PC were decreased in the *ptTES1* knockout mutant when compared to WT (Fig. 5c). As a control, transformation of the empty plasmid pPha-T1 or pTH into WT had no influence on cell growth rate, fatty acid composition and TAG content under the same culture conditions (data not shown).

**Fig. 6:**
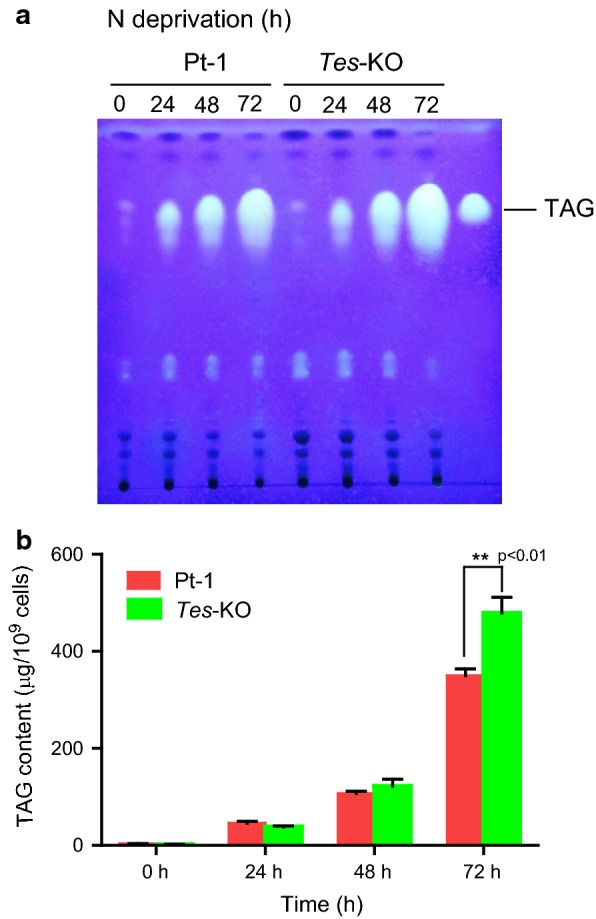
*P. tricornutum* wild-type and ptTES1 knockout line *Tes*-KO were grown in nitrogen-deprived F/2 media for 0, 24, 48, and 72 h. Algal cells were harvested at each time point and total lipids were extracted for TLC analysis (**a**) and determination of TAG content (**b**). Data are expressed as mean ± SD (*n *= 3). Asterisks indicate the significant difference compared with WT (*t* test, **P < 0.01)

WT *P. tricornutum* cells usually accumulate large amounts of TAGs under nitrogen-limited condition. To further test the effect of ptTES1 knockout on nutrient limitation-induced TAG accumulation, we compared WT with *Tes*-KO strain after transfer into nitrogen-deprived F/2 media to induce lipid accumulation. TAGs were isolated from total lipids by thin-layer chromatography (TLC) and the amounts were determined over a time course of 72 h. As shown in Fig. 6a, the amounts of TAGs in both WT and *Tes*-KO strains were gradually increased during nitrogen deprivation and reached the maximum at 72 h. After 72 h of nitrogen deprivation, *Tes*-KO contained significantly more TAGs per cell than WT (Fig. 6b). An interesting finding is that nitrogen deprivation inhibited the growth of both strains, but *Tes*-KO exhibited a higher growth rate than WT during the time course of nitrogen deprivation. *Tes*-KO therefore had a significantly higher TAG titer than WT.

**Fig. 7:**
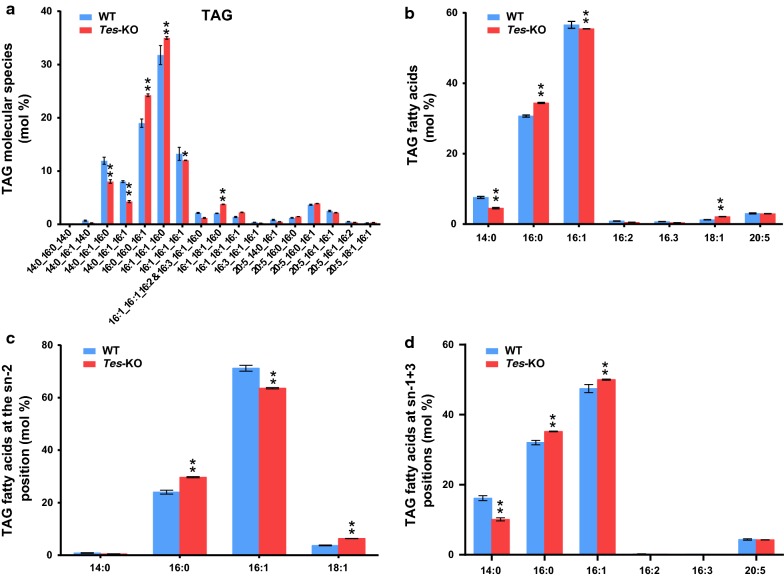
Analysis of triacylglycerols from *P. tricornutum* wild type and the *Tes*-KO mutant grown in nitrogen-replete F/2 media for 8 days. Change of the molecular species composition of TAG (**a**), fatty acid distribution in TAG (**b**), the distribution of fatty acids at *sn*-2 (**c**) and *sn*-1 + 3 positions (**d**) of TAG in *P. tricornutum* strains. Data are the means of three experimental replicates with SD. Asterisks indicate statistically significant differences from WT based on Student’s *t* test (*P < 0.05, **P < 0.01). *TAG* triacylglycerol

To further obtain the detailed information on glycerolipid profiles, we analyzed different molecular species of polar glycerolipid classes in both WT and *Tes*-KO cells (Additional file 3: Figure S3). Of the molecular species of polar lipid classes, 20:5/16:3-MGDG, 14:0/16:1-SQDG, 20:5/16:1-PC, and 20:5/16:1-DGDG showed obvious decrease, while 20:5/16:4-MGDG, 20:5/16:0-SQDG, 16:1/18:1-PC, 20:5/18:2-PC, 16:1/16:0-DGDG and 16:1/16:1-DGDG showed an increase in *Tes*-KO cells when compared to that in WT cells (Additional file 3: Figure S3A–D). In contrast to most phospholipid classes, TAGs were dominated by the molecular species enriched with C14 and C16 saturated and monounsaturated fatty acids. In *Phaeodactylum*, 16:0/16:0/16:1 and 16:1/16:1/16:0 are the most abundant molecular species of TAG, and the levels of both of them are significantly increased in the *ptTES1* knockout mutant when compared to that of WT. In addition, we observed a decrease in the levels of 14:0/16:1/16:0 and 14:0/16:1/16:1 of TAG isolated from the mutant (Fig. 7a). As shown in Fig. 7b, TAG isolated from *Phaeodactylum* was enriched in C16 saturated and monosaturated fatty acids. There was an increase in 16:0 at the expense of 14:0 in TAG from the *ptTES1* knockout mutant when compared to that from WT. We further carried out the stereo-specific analysis of fatty acid distribution in TAG from *Phaeodactylum* and found that more than 90% of acyl chains at the *sn*-2 position of TAG are 16-carbon fatty acids. In addition, 16:0 was present at a higher level at both *sn*-2 and *sn*-1 + 3 positions in TAG isolated from the mutant than that from WT, while 16:1 showed a decreased level at the *sn*-2 position in TAG isolated from the mutant (Fig. 7c, d). For *sn*-1/*sn*-3, the relative content of 14:0 was decreased in the *ptTES1* mutant compared to that in WT. This was also observed in the total composition of all TAG acyl groups (Fig. 7b). In view of a 1.7-fold increase of TAG in the *ptTES1* mutant, the increased distribution of 16:0 across all three positions of TAG may suggest that this plastidic fatty acid serves as an important precursor for supply of the enhanced TAG in the mutant.

### Changes of acyl-CoAs

**Fig. 8:**
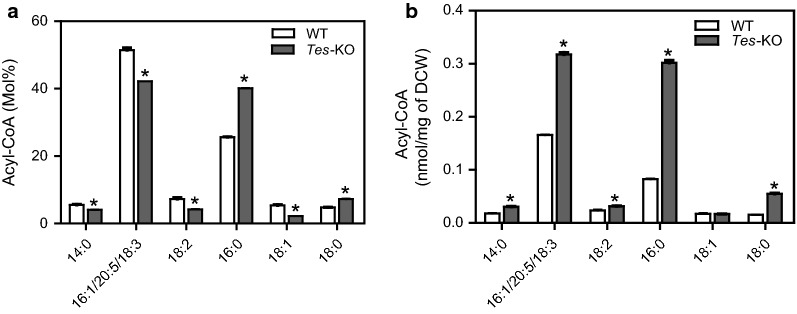
The effect of disruption of acyl-CoA thioesterase gene (*ptTES1*) on the acyl-CoA pool of *P. tricornutum.* Acyl-CoA profile (**a**) and total acyl-CoA concentrations (**b**) of Pt_WT and *ptTES1* knockout line *Tes*-KO cultures grown in nitrogen-deprived F/2 medium for 72 h. Values are the average of three experiments (± SD). Asterisks indicate statistically significant differences from WT based on Student’s *t* test (*P < 0.05)

Because ptTES1 has acyl-CoA thioesterase activity toward various acyl-CoAs with different acyl chain length and saturation in in vitro assay, we compared the acyl-CoA pool composition of wild type and *ptTES1* knockout mutant grown on N-depleted F/2 medium for 72 h (Fig. 8). In our HPLC analysis, C16:1-CoA could not be distinguished from C20:5- and C18:3-CoAs due to the overlapping of detection peak of these three acyl-CoAs. In both WT and mutant, palmitic (C16:0) CoA and the acyl-CoA mixture including C16:1/C20:5/C18:3 CoAs were the major components of the acyl-CoA pool. This is consistent with the predominance of C16:0, C16:1 and C20:5 acids in lipid fatty acid composition (Fig. 5a). Upon N deprivation for 72 h, 25 mol% C16:0-CoA and 5 mol% C18:0-CoA in wild type were increased to 40 mol% and 7 mol% in the *ptTES1* mutant, respectively. The parallel increase of C16:0 in TAG at the *sn*-1 and *sn*-2 + 3 position reported above (Fig. 7b–d) is consistent with the fact that all acyltransferases involved in the de novo TAG synthesis via the Kennedy pathway (glycerol phosphate acyltransferase, GPAT; acylglycerolphosphate acyltransferase, AGPAT and/or lysophosphatidic acid acyltransferase, LPAAT; diacylglycerol acyltransferase, DGAT) might use the increased C16:0-CoA pool in the *ptTES1* mutant. When compared to wild-type, 5 mol% C18:1-CoA, 7 mol% C18:2-CoA, and 51 mol% C16:1/C20:5/C18:3 CoAs were decreased to 2 mol%, 4 mol%, and 42 mol%, respectively (Fig. 8a). The analysis reveals an altered acyl-CoA composition for *ptTES1* inactivation with a marked increase in saturated C16 and C18 acyl-CoAs. The total quantitative acyl-CoA pool sizes, on a dry cell weight basis, for both wild type and *ptTES1* mutant grown on N-depleted medium for 72 h are shown in Fig. 8b. The total acyl-CoA pool size was significantly higher in the *ptTES1* mutant than wild type, which is consistent with more lipid accumulation in the *ptTES1* mutant when algal cells were exposed to N deprivation for 72 h (Fig. 6a). Moreover, while almost no change in acyl-CoA content was observed for C18:1- and C18:2-CoAs, the content of C18:0, C16:0, and C16:1/C20:5/C18:3 CoAs was significantly increased. As C16:0 and C16:1/C20:5/C18:3 CoAs are the major components of the acyl-CoA pool in *P. tricornutum*, significant increase in these acyl-CoAs contributes to the overall elevation of the total acyl-CoA pool size.

## Discussion

In eukaryotic microalgae, heterologous expression or overexpression of key enzymes, such as malic enzyme [7], glycerol-3-phosphate acyltransferase [8], and diacylglycerol acyltransferase [6], has proven to be an efficient strategy to enhance lipid accumulation. In this study, we demonstrate that inactivation of a thioesterase gene involved in acyl-CoA hydrolysis could lead to a significant increase in TAG accumulation. A similar increase in lipid accumulation was also observed in *P. tricornutum* by inactivation of the UDP-glucose pyrophosphorylase gene [11], and in *Chlamydomonas reinhardtii* by mutation of the ADP-glucose pyrophosphorylase gene [29]. Although an increase in lipid accumulation was achieved in these examples, the underlying mechanism or the used strategy may be different. In our study, acyl-CoA availability was increased by blocking of ptTES1-catalyzed acyl-CoA hydrolysis, while the others demonstrated the carbon flux or allocation from storage of polysaccharides or starch toward lipid accumulation. These competing pathways are present within cells and have a crucial impact on the quantity and composition of the acyl-CoA pool. Some metabolic engineering examples also demonstrate the positive effect of inhibiting the hydrolysis or degradation of substrate on enhancing the levels of synthetic end products. When the 4-hydroxycoumarin (4HC) synthetic pathway was reconstituted in *E. coli* for metabolic engineering purpose, a thioester intermediate salicoyl-CoA may encounter an undesirable hydrolysis due to the host’s endogenous thioesterase activity. Inactivation of the responsible thioesterase (YdiI) gene led to 300% increase in 4HC production through alleviating the undesirable degradation of thioester intermediates [30].

In the plastids of eukaryotic microalgae including diatom, de novo fatty acid biosynthesis takes place by action of a type II fatty acid synthase (FASII), which is characterized by a multi-domain enzymatic complex and each activity is encoded by a separate protein. Unlike *E. coli*, which normally does not accumulate free fatty acids as intermediates in lipid biosynthesis [31], most microalgae or plants naturally synthesize significant quantity of free fatty acids (FFAs) that are further converted to acyl-CoA thioesters for lipid biosynthesis and accumulation. Intracellular accumulation of FFAs in *E. coli* can be achieved by heterologous expression of acyl-ACP thioesterase, which interrupts the fatty acid elongation cycle and then releases FFAs. In this study, we detected an acyl-ACP thioesterase activity of ptTES1 through the examination of agar plate-based color change caused by ptTES1-mediated FFA release (Fig. 1a); we also verified that ptTES1 has acyl-CoA thioesterase activity through in vitro enzyme assay using various acyl-CoAs as substrates (Fig. 1b–d). Because ACP (a small acidic protein) and coenzyme A (an adenosine-3′-phosphate derivative) have different structural properties, it might be surprising that the same enzyme could recognize both acyl carriers. Similar to *P. tricornutum* ptTES1, plant acyl-ACP thioesterases also have been showed to be active with acyl-CoA-bound substrates [32]. Structural analysis of plant thioesterases reveals a conserved helix/4-stranded sheet architecture, which has been identified among enzymes recognizing either acyl-CoA or acyl-ACP-bound substrates [33]. Algal thioesterases may be distinct from plant acyl-ACP thioesterases, which are classified into two major types of thioesterases (FatA and FatB) and their substrate specificity plays an important role in controlling the fatty acid composition of seed oils [34]. In the green microalga *Chlamydomonas reinhardtii*, a unique thioesterase (CrTE) with both plant FatA and FatB character was demonstrated to interact with chloroplastic ACP, and this significant ACP–TE interaction and substrate recognition mediate the hydrolysis of fatty acids within the algal chloroplast [35]. CrTE can act on several acyl-ACP substrates, and *Phaeodactylum* ptTES1 also shows the hydrolytic activity on various saturated and unsaturated acyl-CoAs, suggesting the promiscuity in substrate specificities of these algal thioesterases.

In spite of this possibility to also act on acyl-ACP, it seems more likely that acyl-CoA is the physiological substrate of ptTES1, supported by the following evidence: (i) ptTES1 shows in vitro hydrolytic activity toward numerous acyl-CoAs, which are endogenous acyl-CoA species detected using the HPLC method in *Phaeodactylum* cells; (ii) inactivation of ptTES1 gene increased the size of the acyl-CoA pool within the *Phaeodactylum* cells; (iii) inactivation of ptTES1 gene also led to a decrease in endogenous FFAs (data not shown), which may be partially derived from thioesterase activity within the cell.

**Fig. 9:**
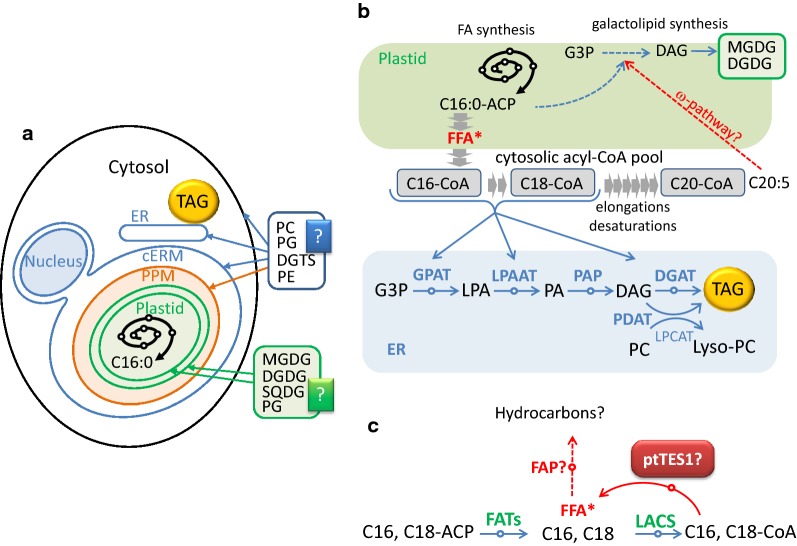
The proposed pathway for TAG biosynthesis and fatty acyl flux in nitrogen-deprived *P. tricornutum*. **a** Cell compartments in *Phaeodactylum.* **b** Cytosolic pool of acyl-CoA in *Phaeodactylum.* **c** Possible role of ptTES1 in free fatty acid homeostasis in the plastid. See text for details. *cERM* chloroplast endoplasmic reticulum membrane, *DAG* diacylglycerol, *DGAT* acyl-CoA:diacylglycerol acyltransferase, *DGDG* digalactosyldiacylglycerol, *FATs* acyl-ACP thioesterases, *FAP* fatty acid photodecarboxylase, *G3P* glycerol-3-phosphate, *GPAT* glycerol-3-phosphate acyltransferase, *LACS* long-chain acyl-CoA synthetase, *LPAAT* lysophosphatidic acid acyltransferase, *LPA* lysophosphatidic acid, *Lyso-PC* lysophosphatidylcholine, *LPCAT* lysophosphatidylcholine acyltransferase, *MGDG* monogalactosyldiacylglycerol, *PA* phosphatidic acid, *PAP* phosphatidic acid phosphatase, *PC* phosphatidylcholine, *PE* phosphatidylethanolamine, *PG* phosphatidylglycerol, *PPM* periplastidial membrane, *PDAT* phospholipid:diacylglycerol acyltransferase, *SQDG* sulfoquinovosyl diacylglycerol, *TAG* triacylglycerol

Plant acyl-ACP thioesterases are nuclear encoded, but plastid-targeted proteins. In our study, although a little GFP fluorescence can be observed in the cytosol near the plastid for the *P. tricornutum* transformants expressing ptTES1–eGFP fusion protein (Fig. 2), ptTES1 was identified unambiguously and predominantly as a plastidial enzyme and therefore is likely to function in a plastidial process like fatty acid synthesis. The identification of a putative mitochondrial transit peptide could suggest a dual targeting to the mitochondrion as well, although we did not detect such localization of the eGFP fusion protein, supporting a possible multifunctionality of this thioesterase. Based on the plastidial localization of ptTES1 and its thioesterase activity toward various acyl-CoAs, we hypothesized that ptTES1 might function in the hydrolysis of acyl-CoAs in *Phaeodactylum* plastid. Unlike plants containing primary plastids, *Phaeodactylum* has a secondary plastid which is surrounded by four membranes and thus there are four different compartments (Fig. 9a). In the plastidial fatty acid biosynthesis, 16:0-ACP and 16:1-ACP are initially produced by FASII and the palmitoyl-ACP desaturase (PAD) in the stroma, and then they are exported to the cytosol after a conversion into FFA. In plant cells containing primary plastids, FFAs are activated into acyl-CoAs upon reaching the cytosol by LACS enzymes (Fig. 9b), feeding glycerolipid metabolism, including TAG biosynthesis. It is possible that a supply of acyl-CoA exists in the chloroplast endoplasmic reticulum membrane (cERM) or in the periplastid compartment membrane (PPM) of the secondary plastid of *Phaeodactylum* (Fig. 9). An FFA→acyl-CoA→FFA cycle might be one of the mechanisms controlling FFA crossing the four plastid membranes, although FFAs can also been exported out of the plastid through free crossing (Fig. 9b, c).

Our results are consistent with a role of pTES1 in the control of free fatty acyl transfers outside and inside the chloroplast. On the one hand, if pTES1 was involved in the export of C16:0 and/or C16:1 (Fig. 9c), an increase in the proportion of these fatty acids in plastid should be observed in ptTES1-inactivated mutant. Indeed, there is an increase in the molecular species of MGDG, DGDG and SQDG containing 16:0/16:0, 16:1/16:0 or 16:1/16:1 in the lipidome of ptTES1-inactivated mutant when compared to the wild-type strain (Additional file 3: Figure S3). In the cytosol, the flux of 16:0 is crucial for synthesizing 20:5. On the other hand, changes in FA profiles in glycerolipid classes also support a role of ptTES1 in FFA import, mainly of C20:5. Although the actual mechanism by which C20:5 is reintroduced into the plastid, via the so-called ω-pathway [36], has not been elucidated, this polyunsaturated fatty acid is likely to be imported as FFA. We observed a decrease in the proportion of C20:5 in MGDG, DGDG and SQDG in ptTES1-inactivated mutant, which supports a role of ptTES1 in the occurrence of C20:5. The decreased accumulation of C20:5 in MGDG is expected to have an impact on the balance between the syntheses of some glycerolipid classes. As reported in *Nannochloropsis*, C16:0 can be the entry point into a channeled production of 20:5 via Δ0-ELOs [12]. Blocking this process or downstream processes should lead to a direct decrease in MGDG content and a diversion of excess 16:0 toward TAG biosynthesis. In the *pTES1* knockout mutant, if C20:5 is prevented from reaching the plastid, MGDG overall quantity should decrease (Additional file 3: Figure S3), then following the mass action law, some upstream intermediates for 20:5 should accumulate, such as 18:1 and 18:2 in PC (Additional file 3: Figure S3), and most importantly the excess flow of 16:0 should be directed toward TAG synthesis. As shown in Fig. 6b, a significant increase in TAG content can be observed in *ptTES1* knockout mutant.

Overall, the change in the lipidomic profile observed in the pTES1 knockout mutant is consistent with a (or partial) role of this thioesterase in the crossing of the plastid membranes by fatty acids, i.e., the export of 16:0 and 16:1 and the import of 20:5. Given the critical role of 16:0 as the basal precursor for the import of 20:5, this ptTES1-mediated acyl flux is supposed to be important in glycerolipid synthesis. In the *ptTES1* knockout mutant, intracellular acyl-CoA content was increased. It is likely that the enhanced acyl-CoAs are utilized as substrate by the conventional TAG biosynthesis pathway, which involves sequential transfer of acyl chains to glycerol backbone in the endoplasmic reticulum (ER). Alternatively, acyl groups of the enhanced acyl-CoAs may also be incorporated into phosphatidylcholine (PC) through a deacylation/reacylation cycle, in which acyl-CoA:lysophosphatidylcholine acyltransferase (LPCAT) plays a central role [37, 38]. The resulting PC can be converted to DAG for TAG synthesis or transacylated to form TAG by phospholipid:diacylglycerol acyltransferase (PDAT) [39]. These pathways/enzymes may use the enhanced acyl-CoAs as substrates and thus may be responsible for the increased TAG synthesis in the *ptTES1* mutant.

In addition, it has been recently reported that FFA could be converted into hydrocarbons in microalgae via the action of an FA photolyase (FAT) [40]. A FAT homolog is present in *Phaeodactylum* (Phatr3_J1341). The magnitude of the conversion of FFA into hydrocarbons and the role of the FAT enzyme in *Phaeodactylum* are not known; however, ptTES1 might control the level of FAT substrate as well (Fig. 9c).

The presence of acyl-CoA thioesterase activity of ptTES1 raised the question of which enzyme(s) catalyzes the further esterification of FFA produced by the action of ptTES1 in the plastid. In our previous study, we identified five long-chain acyl-CoA synthetases (LACS) in the diatom *Phaeodactylum* [41]. We recently found that at least four LACS enzymes are targeted into the plastid (unpublished data), supporting the possibility that plastid-associated ptTES1 generates FFAs that become substrates for certain LACS enzyme(s). This would provide an alternative route to plastidial ptTES1 for trafficking of FFAs to the plastidial LACS enzyme(s), and this route should therefore control carbon flux toward TAG biosynthesis in *P. tricornutum* cells.

We observed that the *ptTES1*-KO mutant grew at a slower rate than wild-type strain during the late growth phase and that the enhanced lipid accumulation occurred synchronously when the *ptTES1*-KO mutant began to grow at a reduced rate compared to the wild-type strain (Fig. 4). Measurement of several photosynthetic parameters, including *Fv*/*Fm*, qP, ETR and chlorophyll content, showed that WT and the *ptTES1* mutant had similar photosynthetic performance (data not shown). Moreover, there is also no difference of nitrogen consumption rates between WT and the mutant (Fig. 4b). The differential growth rates and lipid accumulation observed in WT and the mutant might be correlated to other biochemical activities such as the central carbon metabolism. During nitrogen deprivation, the *ptTES1* knockout mutant exhibited a higher growth rate than WT, suggesting that nitrogen deprivation had a less influence on the growth of the mutant than on that of WT. This allows enhanced lipid productivity achieved in the *ptTES1* knockout mutant in comparison to WT. In large-scale production of algae-derived lipids, the two-stage cultivation is always a method that combines distinct growth stages in different culture systems. In the first stage, microalgae are always grown in nutrient-replete media that favor continuous cell division. The second production stage is aimed at exposing algal cells to nutrient stresses, which greatly enhance lipid synthesis [42]. Application of this two-stage production process for cultivation of the engineered algal strain might help achieve the optimum lipid productivity in large-scale production of biofuels.

## Conclusions

This work suggests that a Hotdog-fold thioesterase localized in the plastid of the model diatom *Phaeodactylum* exhibits in vitro hydrolytic activity toward a wide range of saturated and unsaturated fatty acyl-CoAs ranging from C10 to C20, and shows the highest activity with C18:0-CoA. Disruption of this acyl-CoA thioesterase gene using TALEN-based genome editing technique resulted in a significant increase in TAG content, but almost no change of other phosphoglycerolipids and galactoglycerolipids. The biochemical analyses of ptTES1 enzyme, combined with the phenotypic analyses of *Phaeodactylum* overexpressor and mutant lines, are consistent with a multifunctional role of ptTES1 in FFA homeostasis in the plastid. Nitrogen deprivation further enhanced the TAG content and titer of the engineered algal strain, which achieved markedly higher TAG productivity under nitrogen deprivation for 72 h in comparison to the wild-type strain. Importantly, this enhanced lipid-producing property of the engineered algal strain could be maintained for more than 1 year. We believe that the use of TALEN-based genome editing technique enables knowledge-based studies of genes involving lipid metabolism and also demonstrates the potential of engineering of diatoms to produce biofuels and other high-value fatty acid-derived chemicals.

## Methods

### Strains and growth conditions

*Escherichia coli* strains were grown on Luria–Bertani broth or agar supplemented with ampicillin (100 mg/L) or kanamycin (50 mg/L) as needed. *P. tricornutum* Pt1 wild-type and knockout strains were grown in F/2 medium [43] at 22 °C under a 16-h light/8-h dark regime with 50 μmol photons m^−2^ s^−1^ white light. The algal culture was bubbled with filtered air. Zeocin (50 mg/L) was supplemented when the *P. tricornutum* gene knockout mutant was grown in F/2 medium.

For the growth experiment of wild-type and knockout strains, algal cultures were inoculated with 2 × 10^5^ cells/mL and grown under continuous illumination of 60 μmol photons m^−2^ s^−1^ on a shaking table with continuous shaking at 60 rpm. Sampling was performed every 2 days for determination of cell number, nitrate concentration and neutral lipid content. Nitrate concentration in the medium was measured spectrophotometrically at 220 nm [44]. The relative neutral lipid content was monitored by fluorometric assay using the dye Nile Red (Sigma-Aldrich). A total of 6 × 10^6^ algal cells for each sample were stained with Nile Red and the fluorescence was measured at 572 nm using an F-7000 Fluorescence Spectrophotometer (HITACHI, Japan) as described by Ge et al. [45].

### Plasmid construction

*Escherichia coli* DH5α (Transgen, China) was used for plasmid construction and propagation according to the manufacturer’s instructions. To functionally express ptTES1 in *E. coli* XL1-blue, the codon-optimized ptTES1 gene was synthesized and cloned in frame into pUC18 (Takara, China), generating the pUC18-phaeoTES1 plasmid. To overexpress ptTES1 in *E. coli* Rosetta (DE3), the codon-optimized ptTES1 gene was synthesized and cloned into pET28a (Novagen), generating the pET28a-phaeoTES1 plasmid. All primers used in this study are listed in Additional file 4: Table S1, and all constructs were confirmed by DNA sequencing.

### Protein expression and purification in *E. coli*

Freshly inoculated *E. coli* strain harboring pET28a-phaeoTES1 was grown in LB broth containing 50 μg/mL kanamycin to an optical density of ~ 0.6 at 600 nm, and then induced by the addition of 0.5 mM IPTG (isopropyl-β-d-thiogalactopyranoside) at 28 °C for 4 h. Cells were harvested by centrifugation at 7000×*g* for 8 min and stored at − 20 °C. Frozen cells were thawed on ice and resolved in buffer A (25 mM Tris–HCl [pH 7.4], 100 mM NaCl, and 1 mM PMSF) and lysed by sonication on ice (5 s on/5 s off cycles for a total time of 10 min). Cell debris was pelleted at ~ 10,000×*g* for 8 min, and the supernatant was loaded onto a 5 mL nickel–nitrilotriacetic acid (Ni–NTA) agarose column (Millipore). Nonspecific proteins were removed by washing with 50 mL of buffer B (25 mM Tris–HCl [pH 7.4], 500 mM NaCl, and 10 mM imidazole), and ptTES1-His6 protein was eluted with 20 mL of buffer C (25 mM Tris–HCl [pH 7.4], 500 mM NaCl, and 500 mM imidazole). The fractions were transferred to a 10 kDa molecular weight cutoff Amicon Ultra-15 centrifugal filter unit (EMD Millipore) for concentration and buffer exchange into 25 mM Tris–HCl (pH 7.4). Glycerol was added to a final concentration of 5%, and the purified protein was stored at − 20 °C. Protein purity was assessed on 12% SDS-PAGE (sodium dodecyl sulfate-polyacrylamide gel electrophoresis) gels and protein concentration was measured using the BCA method (TIANGEN, China).

### Acyl-CoA thioesterase enzyme assay

Thioesterase-catalyzed hydrolysis of acyl-CoA molecules generates free CoA, which can react with 5,5′-dithiobis(2-nitrobenzoic acid) (DTNB) to form 5-thio-2-nitrobenzoate. This product has a maximum absorbance at 412 nm (*A*412) with a molar absorption coefficient of 13.6 mM^−1^ cm^−1^ [46]. Acyl-CoA thioesterase activity of purified ptTES1 was measured spectrophotometrically by monitoring the increase in *A*412 as previously described [19, 47]. Briefly, each assay contained 50 mM KCl, 10 mM HEPES (pH7.5), 0.3 mM DTNB and appropriate amount of decanoyl-CoA. Reactions were carried out in 200 μL volumes within the wells of 96-well plates (Bio-Rad) at room temperature. The reaction was initiated by the addition of recombinant ptTES1-His_6_ (~ 25 μg) to a final volume of 200 μL. The plates were immediately loaded into a SpectraMax M2 microplate reader (Molecule Devices), mixed for 5 s and the absorbance at 412 nm was read at 1-min intervals for 160 or 240 min. Acyl-CoAs of various carbon chain lengths and saturations were obtained from Sigma-Aldrich. One unit of thioesterase activity is defined as the amount of enzyme required to hydrolyze 1 mmol of substrate per min.

### Assembly of TALEN plasmids

To specifically knockout ptTES1, TALEN-based insertional mutagenesis was used. The target site within the *P. tricornutum ptTES1* gene (JGI Protein ID 33198; Ensembl gene reference Phatr3_J33198) was generated using the TAL Effector Nucleotide Targeter 2.0 (https://tale-nt.cac.cornell.edu/node/add/talen) [48] with a fixed repeat array length of 19 per TALEN (the first NG is not counted by the software), a spacer length of 19 bp between the two TALENs. A TALEN binding pair was chosen from the coding sequence of ptTES1 in an exon region in or upstream of the predicted 4HBT_2 domain. The genomic recognition sequences of TALEN left and right arms are TGACGTGGATTACCTCGGA (L) and TAACTCTGCATGGGATAAAT (R), separated by a 19-nt spacer and anchored by a preceding T base at the − 1 position to meet the optimal criteria for natural TAL proteins [49]. TALEN vectors of left and right arms, TALEN-ptTES1-L10 and TALEN-ptTES1-R5, were obtained by one-step ligation using the FastTALE™ TALEN Assembly Kit (SIDANSAI Biotechnology, China) according to the manufacturer’s instructions. The resulting plasmids were used as template to perform DNA sequencing with the primer pair TALEseq-for/TALEseq-rev to verify the correct integration and order of the 20 targeting TALE monomers. To assemble the upstream and downstream TALENs into the *P. tricornutum* expression vector pTH, the sequence-verified TALENs were amplified by PCR with PrimeSTAR^®^ HS DNA Polymerase (TaKaRa, China). The primer pair Talen-tes1en-1/Talen-tes1en-2 was used to amplify the upstream TALEN and Talen-tes1en-3/Talen-tes1en-4 for the downstream TALEN. PCR products were purified using the MiniBEST Agarose Gel DNA Extraction Kit (TaKaRa, China). The purified DNA products were assembled by the EasyGeno Assembly Cloning Kit (TIANGEN, China) with pTH vector digested with I-SceI and I-CeuI. Digestion of the pTH vector with these two restriction enzymes generates two linearized vectors, both of which were used in the assembly to insert the upstream TALEN at the I-CeuI site and the downstream TALEN at the I-SceI site by efficient recombination between 20 nt homologous regions at the linear vector and insert fragments. After the assembly, the reaction mixture was transformed into *E. coli* DH5α and the positive colonies were selected by PCR. The resulting plasmid contains the two assembled TALENs (pTH-ptTES1-TALENs) driven by the *FcpB* or *FcpF* promoter. To construct the KO-plasmid containing the ShBle expression cassette which is flanked by homology regions, primer pairs Tes1up-easy-1/Tes1up-easy-2 and Tes1dw-easy-1/Tes1dw-easy-2 were used to amplify the homology sequences located upstream (ptTES1Up) and downstream (ptTES1Dn) of the TALEN recognition sequence, respectively. The amplified ptTES1-Up was first purified using the MiniBEST Agarose Gel DNA Extraction Kit (TaKaRa, China), then assembled using the EasyGeno Assembly Cloning Kit with the pPha-ShBle-KO19F vector which was linearized with *Xba*I, resulting in the plasmid of pPha-ptTES1Up-ShBle-KO19F. To insert ptTES1Dn immediately downstream of the *ShBle* resistance gene, purified ptTES1Dn was assembled using the same method with pPha-ptTES1Up-ShBle-KO19F which was linearized with *Xho*I, resulting in the plasmid of pPha-ptTES1Up-ShBle-ptTES1Dn-KO19F. This KO-plasmid and the pTH-ptTES1-TALENs plasmid were co-transformed into *P. tricornutum* by microparticle bombardment.

### Nuclear transformation of *P. tricornutum*

Nuclear transformation of *P. tricornutum* was performed using a Bio-Rad Biolistic PDS-1000/He Particle Delivery System (Bio-Rad, Hercules, CA, USA) fitted with 1100/1350 psi rupture disks as described by Zaslavskaia et al. [50]. Approximately, 10^8^ cells per plate were bombarded with 1.5 μg of each plasmid. Bombarded cells were illuminated for 24 h prior to resuspension in 1 mL of sterile seawater and 300 μL (approx. 3 × 10^7^ cells) was spread on fresh F/2 agar medium containing 75 μg/mL Zeocin (Invitrogen, Carlsbad, CA, USA). The plates were placed under constant illumination (75 μΕ/m^2^/s) for 2–3 weeks.

### Generation of the *ptTES1* knockout mutant

Approximately, 30 colonies appeared for the cobombardment of KO- and pTH-ptTES1-TALEN plasmids. These colonies were restreaked on fresh 1.2% F/2 agar plates containing 100 μg/mL Zeocin. The resistant colonies grown on agar plates for 7 days were transferred into 5 mL of liquid F/2 medium containing 100 μg/mL Zeocin and grown at 22 °C for an additional 7 days. Genomic DNA of the resistant colonies was isolated from 2 mL of algal culture as described by Falciatore et al. [51]. Primer pairs for PCR amplification using genomic DNA as template include ptTES1KO-for/ptTES1KO-rev to screen for insertion into the *ptTES1* gene and KmR-for/KmR-rev to screen for the presence of the *sh ble* resistance gene.

### Protein isolation and western blotting

For protein extraction, 100 mL of *P. tricornutum* cells (5 × 10^6^) were collected by centrifugation and immediately frozen in liquid nitrogen. Approximately, 1.5 mL of isolation buffer (50 mM Tris HCL, pH 8.0) supplemented with protease inhibitor (Sigma) was added to the frozen pellet. Algal cells were disrupted by ultrasonication on ice for 15 min. Cell debris and unbroken cells were removed by centrifugation for 25 min at 12,000×*g* and at 4 °C. The protein concentration of the supernatant was determined using the BCA Protein Assay Kit (Pierce) according to the manufacturer’s guidelines. Protein samples were subjected to standard SDS-PAGE and transferred to PVDF membranes (GE, Healthcare, Pittsburgh, USA) in 25 mM Tris, 192 mM glycine, and 20% methanol. Immunoblot was performed as described in Chen et al. [52] using α-ptTES1 antibody. The signals were visualized using the method of chemiluminescence detection. For detection of ptTES1 in the plastid, we performed cell fractionation as described by Schober et al. [53]. The isolated plastid of *P. tricornutum* was disrupted by ultrasonication on ice for 15 min to prepare the protein lysate, which was used for further immunoblot analyses.

### Glycerolipid analysis

Glycerolipids were extracted from freeze-dried *P. tricornutum* cells grown in 50 mL of F/2 medium as previously described [54]. The total glycerolipids were quantified from their fatty acids, in a 10 µL aliquot fraction. A known quantity of C15:0 was added and the fatty acids present were transformed as methyl esters (FAME) by 1 h incubation in 3 mL 2.5% H_2_SO_4_ in pure methanol at 100 °C [55]. The reaction was stopped by the addition of 3 mL water, and 3 mL hexane was added for phase separation. After 20 min of incubation, the hexane phase was transferred to a new tube. FAME were extracted a second time via the addition, incubation and extraction of another 3 mL hexane. The combined 6 mL was argon dried and re-suspended in 30 µL hexane for gas chromatography–flame ionization detector (GC–FID) (Perkin Elmer) analysis on a BPX70 (SGE) column. FAME were identified by comparison of their retention times with those of standards (Sigma) and quantified by the surface peak method using C15:0 for calibration. Extraction and quantification were performed with at least three biological replicates. For quantification of specific classes of glycerolipids, including TAG, total lipids were separated by thin layer chromatography (TLC) onto silica gel plates (Merck) as described previously [56]. Glycerolipid molecular species were analyzed by LC–MS/MS. For a technical triplicate analysis, an aliquot of the lipid extract containing 25 nmol of total fatty acid was dried under argon and dissolved in 100 µL of a methanol/chloroform solution (1:2) containing 125 pmol of 18:0/18:0/18:0 TAG as internal standard. For each replicate, 20 µL was injected in a high-performance liquid chromatography coupled to a tandem mass spectrometry (LC–MS/MS) system. The analytic device comprised an LC system with binary pumps (Agilent 1260 Infinity) coupled to a triple quadrupole MS (Agilent 6460) equipped with a JetStream electrospray vane of injection. TAG were separated by LC from other lipids using a diol column (Macherey-Nagel, EC 150/2 Nucleosil 100-5 OH) maintained at 40 °C. The chromatography conditions were as follows: solvent A: isopropanol/water/ammonium acetate 1 M, pH 5.3 (850/125/1); solvent B: hexane/isopropanol/water/ammonium acetate 1 M, pH 5.3 (625/350/24/1); gradient: 0–5 min 100% B, 5–30 min linear increase of A to 100%, 30–45 min 100% A, 45–50 min: linear increase of B to 100%, 50–70 min 100% B. Under these conditions, TAG were eluted after 4–5 min of run. The various TAG species were detected from their *m*/*z* ratio by MS/MS using the multiple reaction monitoring (MRM) mode. The various transition reactions used to identify the different TAG species are those previously established with *P. tricornutum* [44]. Quantification was made as described previously [57] using the Agilent Mass Hunter^®^ software furnished by the MS supplier.

### Extraction and quantitative analysis of acyl-CoAs

Diatom cells were harvested by centrifugation at 4000×*g* for 10 min, frozen in liquid nitrogen and stored at − 80 °C. Acyl-CoAs were extracted from wild type and ptTES1 knockout mutant according to the protocol described by Larson and Graham [58]. For the extraction of acyl-CoAs, 70 mg of lyophilized biomass was homogenized in 1.5 mL of extraction buffer (2-propanol, 1 mL; 50 mM KH_2_PO_4_ pH7.2, 1 mL; acetic acid, 25 μL and 50 mg/mL fatty acid-free BSA). Heptadecanol-CoA (50 ng) was added as an internal standard. The sample was then mixed with 1 mL of petroleum ether saturated with 2-propanol: water (1:1, v/v), and spun for 3 s. The mixture was centrifuged at 2000×*g* for 3 min to obtain good phase separation. The interphase was collected and washed with saturated petroleum ether three times to remove pigments and lipids. The upper phase was removed and the aqueous phase containing the long-chain acyl-CoA esters was mixed with 5 μL of saturated (NH_4_)_2_SO_4_ and 600 μL of methanol: chloroform (2:1, v/v). The mixture was incubated at room temperature for 20 min, followed by centrifugation at 21,000×*g* for 2 min. The supernatant was collected, dried under nitrogen and then re-suspended in 150 μL of derivatization buffer containing 0.5 M chloracetaldehyde, 0.15 M citrate buffer (pH4.0), and 0.5% (w/v) SDS. The derivatization was performed at 85 °C for 20 min, and then the samples were cooled down to room temperature for further analysis. Derived internal standard and sample acyl *etheno* CoA esters were separated by high-performance liquid chromatography (HPLC) using a quaternary gradient system as described by Larson and Graham [58]. Briefly, a 2.1 × 150 mm column with phenyl-coated 5 μm silica particles was used, together with a phenyl guard column (ACE, UK). The HPLC equipment was supplied by Agilent 1260. The mobile phase compositions were as follows: A, 1% acetic acid; B, 90% acetonitrile 1% acetic acid; C, 0.25% triethylamine 0.1% tetrahydrofuran; D, 90% acetonitrile. The gradient elution profile was as follows: 0–5 min, 0.75 mL/min 10% B 90% A to 80% B 20% A; 5–5.1 min, 80% B 20% A to 80% C 20% A; 5.1–7 min, 80% C 20% A to 97% C 3% D; 7–10 min, 97% C 3% D to 95% C 5% D; 10–10.1 min, flow rate reduced to 0.2 mL/min; 10.1–50 min, 95% C 5% D to 55% C 45% D; 50–50.1 min, 55% C 45% D to 100% D; 50.1–52 min, 100% D; 52–52.1 min, flow rate increased to 0.2 mL/min; 52.1–57 min, 100% D; 57–57.1 min, 100% D to 10% B 90% A; 57.1–60 min, 10% B 90% A. Samples were maintained at 15 °C in the autosampler prior to injection of 20 μL volumes, and the column was maintained at 40 °C. Peaks of fluorescent derivatives were detected using a G1321B fluorometer. The excitation wavelength was set to 230 nm and the emission wavelength to 420 nm. The acyl-CoA content was calculated from the peak area of internal standard (heptadecanoyl-CoA) with known concentrations.

## Additional files

**Additional file 1: Figure S1.** Acyl-CoA thioesterase activity of recombinant ptTES1 purified from *E. coli*. The time-dependent CoA release catalyzed by ptTES1 was monitored at *A*_412_ for various substrate concentrations. Reactions were performed at room temperature in mixtures that contain 25 μg recombinant ptTES1 protein, 50 mM KCl, 10 mM HEPES (pH7.5), 0.3 mM DTNB, and various concentrations of acyl-CoA substrates including malonyl-CoA (**A**), 10:0-CoA (**B**), 14:0-CoA (**C**), 16:0-CoA (**D**), 16:1-CoA (**E**), 18:0-CoA (**F**), 18:1-CoA (**G**), and 20:5-CoA (**H**).

**Additional file 2: Figure S2.** Western blotting analysis of protein lysates with α-ptTES1 antibody. The molecular weight marker is labeled on the left. Lane 1, 4 ng of total protein lysate from approx. 1.5 × 10^6^ cells; Lane 2, 4 ng of the protein lysate from the plastid fraction isolated from approx. 6 × 10^8^ cells; Lane 3, 400 ng of the protein lysate from the cytoplasm fraction from approx. 1.3 × 10^6^ cells. Coomassie brilliant blue (CBB) staining is shown as a loading control.

**Additional file 3: Figure S3.** Molar profiles of fatty acids in MGDG (**A**), SQDG (**B**), PC (**C**), and DGDG (**D**) from *P. tricornutum* wild-type and *ptTES1* knockout line *Tes*-KO cells grown in nitrogen-replete F/2 media for 8 days. Values are the average of three experiments (± SD). MGDG, monogalactosyldiacylglycerol; SQDG, sulphoquinovosyl diacylglycerol; PC, phosphatidylcholine; DGDG, digalactosyldiacylglycerol.

**Additional file 4: Table S1.** Strains and primers used in this work.

## Abbreviations

DAG: diacylglycerol
DGAT: acyl-CoA:diacylglycerol acyltransferase
DGDG: digalactosyldiacylglycerol
FAP: fatty acid photodecarboxylase
FATs: acyl-ACP thioesterases
G3P: glycerol-3-phosphate
GPAT: glycerol-3-phosphate acyltransferase
LACS: long-chain acyl-CoA synthetase
LPA: lysophosphatidic acid
LPAAT: lysophosphatidic acid acyltransferase
LPCAT: lysophosphatidylcholine acyltransferase
Lyso-PC: lysophosphatidylcholine
MGDG: monogalactosyldiacylglycerol
PA: phosphatidic acid
PAP: phosphatidic acid phosphatase
PC: phosphatidylcholine
PDAT: phospholipid:diacylglycerol acyltransferase
PE: phosphatidylethanolamine
PG: phosphatidylglycerol
PPM: periplastidial membrane
SQDG: sulfoquinovosyl diacylglycerol
TAG: triacylglycerol
