# Supplementary figures and images for: Enhanced triacylglycerol production in the diatom Phaeodactylum tricornutum by inactivation of a Hotdog-fold thioesterase gene using TALEN-based targeted mutagenesis
Source: Biotechnol Biofuels. 2018 Nov 12;11:312. doi: 10.1186/s13068-018-1309-3 (PMC6231261; doi:10.1186/s13068-018-1309-3)

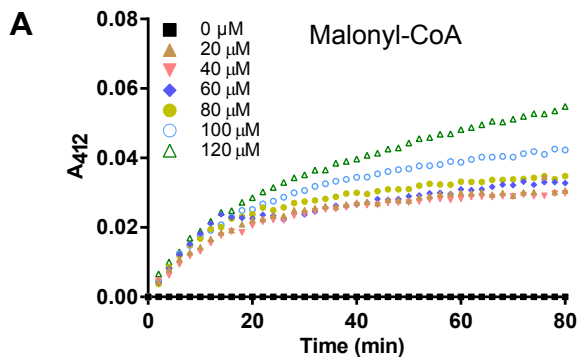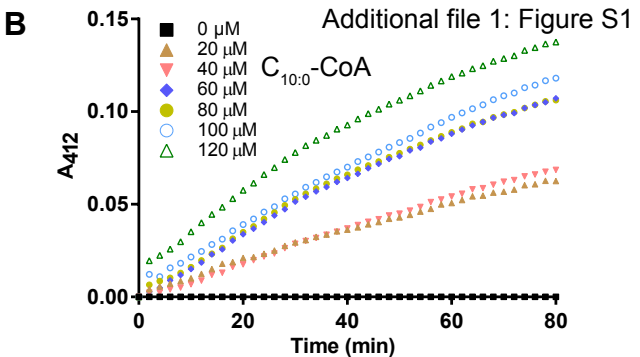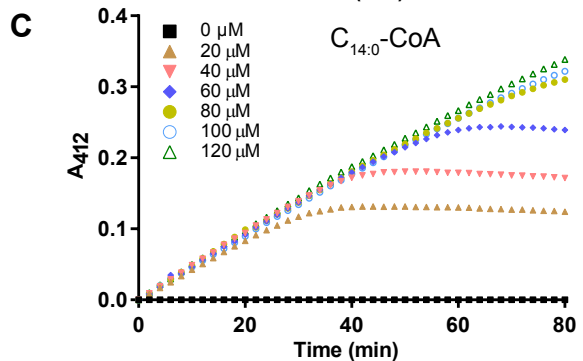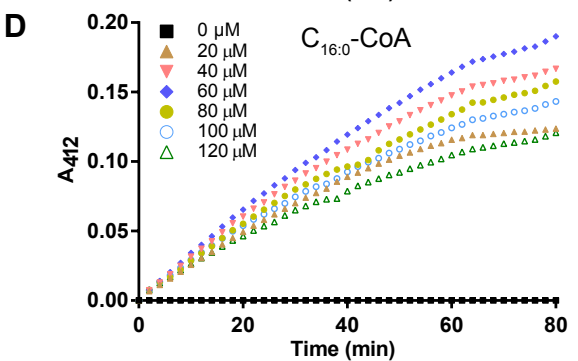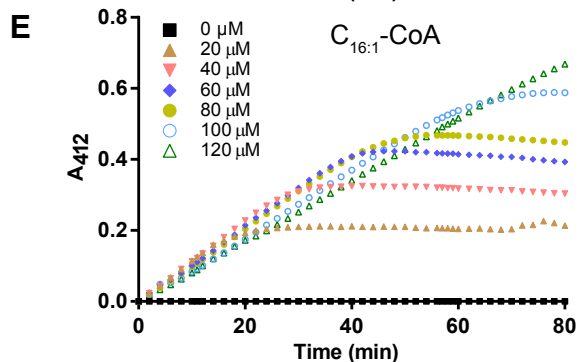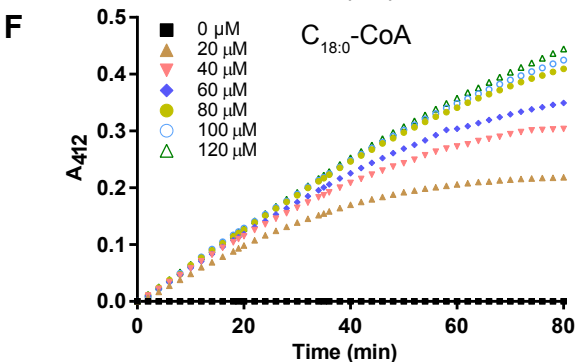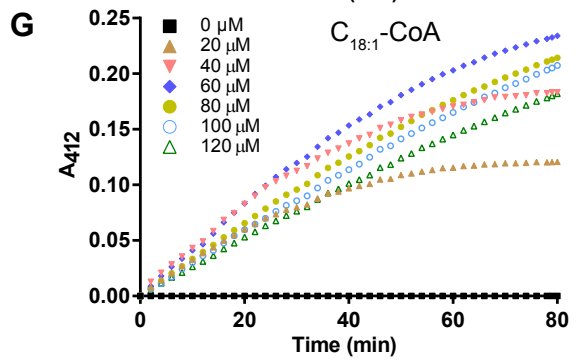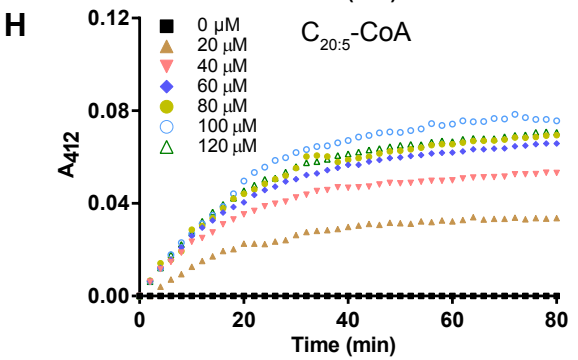

Supplement: Supplementary file 1 — Additional file 1: Figure S1. Acyl-CoA thioesterase activity of recombinant ptTES1 purified from E. coli. The time-dependent CoA release catalyzed by ptTES1 was monitored at A412 for various substrate concentrations. Reactions were performed at room temperature in mixtures that contain 25 μg recombinant ptTES1 protein, 50 mM KCl, 10 mM HEPES (pH7.5), 0.3 mM DTNB, and various concentrations of acyl-CoA substrates including malonyl-CoA (A), 10:0-CoA (B), 14:0-CoA (C), 16:0-CoA (D), 16:1-CoA (E), 18:0-CoA (F), 18:1-CoA (G), and 20:5-CoA (H). [file 13068_2018_1309_MOESM1_ESM.pdf]

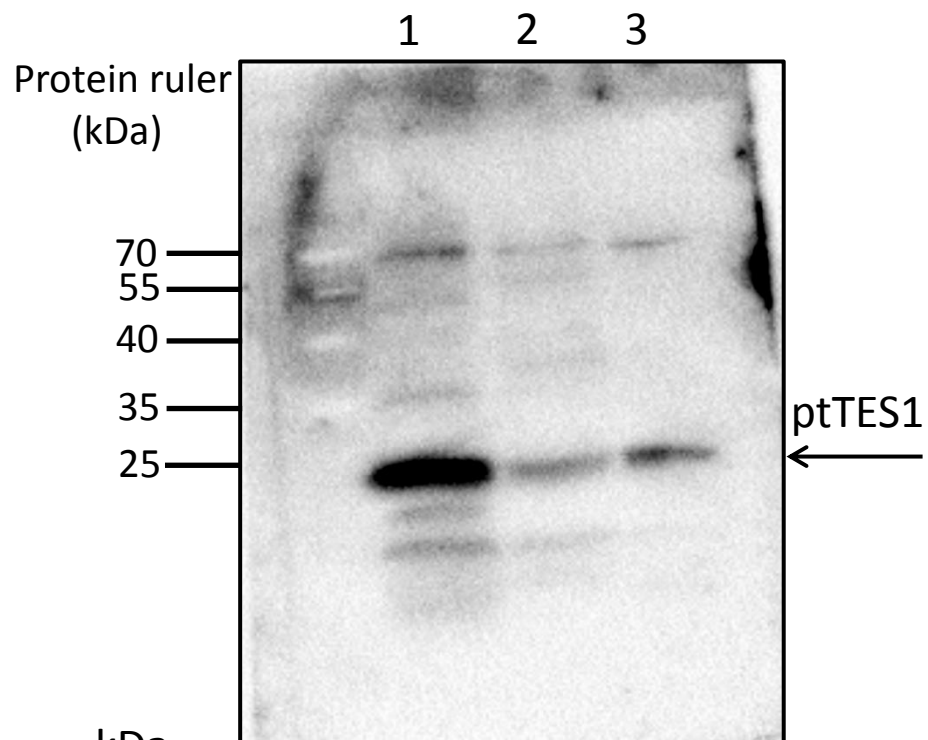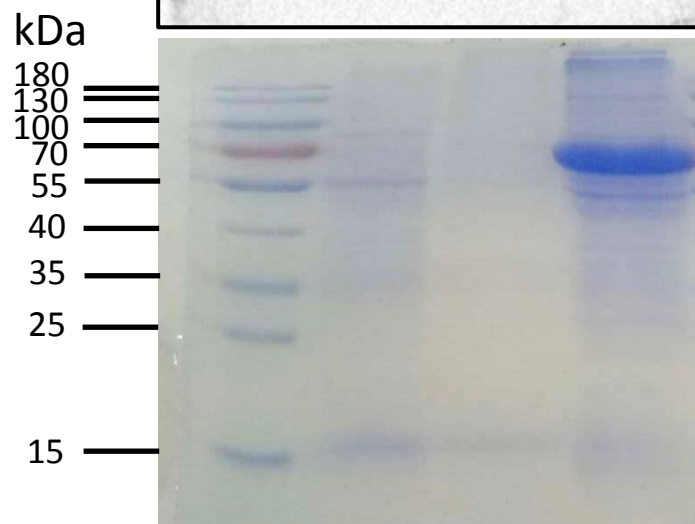

Coomassie Brilliant Blue (CBB)

Supplement: Supplementary file 2 — Additional file 2: Figure S2. Western blotting analysis of protein lysates with α-ptTES1 antibody. The molecular weight marker is labeled on the left. Lane 1, 4 ng of total protein lysate from approx. 1.5 × 10^6^ cells; Lane 2, 4 ng of the protein lysate from the plastid fraction isolated from approx. 6 × 10^8^ cells; Lane 3, 400 ng of the protein lysate from the cytoplasm fraction from approx. 1.3 × 10^6^ cells. Coomassie brilliant blue (CBB) staining is shown as a loading control. [file 13068_2018_1309_MOESM2_ESM.pdf]

**A**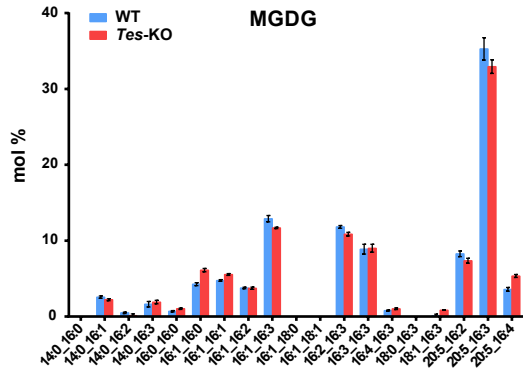**B**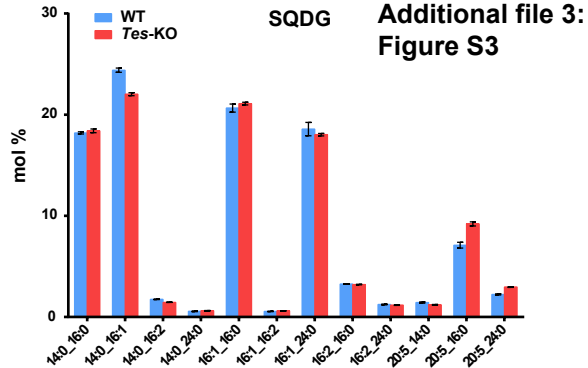**C**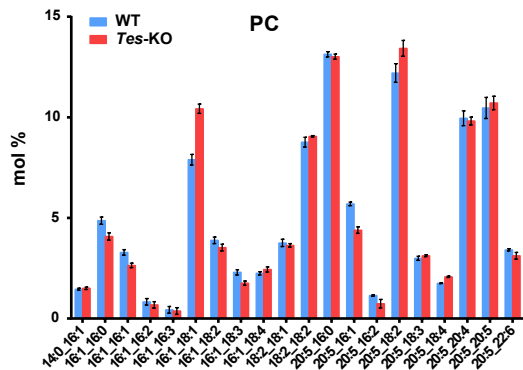**D**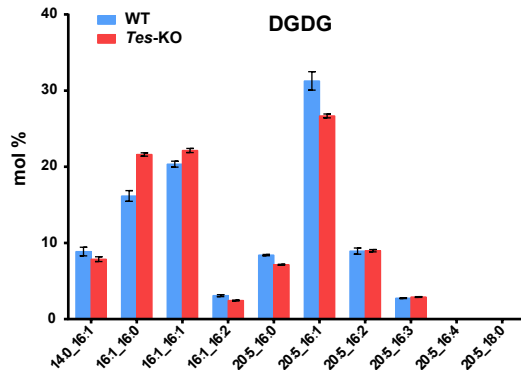

Supplement: Supplementary file 3 — Additional file 3: Figure S3. Molar profiles of fatty acids in MGDG (A), SQDG (B), PC (C), and DGDG (D) from P. tricornutum wild-type and ptTES1 knockout line Tes-KO cells grown in nitrogen-replete F/2 media for 8 days. Values are the average of three experiments (± SD). MGDG, monogalactosyldiacylglycerol; SQDG, sulphoquinovosyl diacylglycerol; PC, phosphatidylcholine; DGDG, digalactosyldiacylglycerol. [file 13068_2018_1309_MOESM3_ESM.pdf]
