# Supplementary material for: Enhanced triacylglycerol production in the diatom Phaeodactylum tricornutum by inactivation of a Hotdog-fold thioesterase gene using TALEN-based targeted mutagenesis
Source: Biotechnol Biofuels. 2018 Nov 12;11:312. doi: 10.1186/s13068-018-1309-3 (PMC6231261; doi:10.1186/s13068-018-1309-3)
Supplement: Supplementary file 4 — Additional file 4: Table S1. Strains and primers used in this work. [file 13068_2018_1309_MOESM4_ESM.pdf]

Table S1 Strains and primers used in this study.

Strains/primers	Use, relevant characteristic (s), and/or sequence (source)
Strains	
E. coli DH5α	*E. coli* host for DNA manipulations, TransGen (Beijing, China)
E. coli Rosetta (DE3)	*E. coli* host for overproduction of recombinant protein
Phaeodactylum tricornutum	Pt1
Primers (5'>3')	
TALEseq-for	CTCCCCTTCAGCTGGACAC
TALEseq-rev	AGCTGGGCCACGATTGAC
ptTES1KO-for	GTCTTCCCAGGCAATCTGC
ptTES1KO-rev	CTTCCAGCGTACTCAACGG
KmR-for	CGTATTTCGTCTCGCTCAGG
KmR-rev	TCGAGCATCAAATGAAACTGC
Talen-tes1en-1	CGTAACTATAACGGTCCTAAGGATGACGATGACAAGATGGC
Talen-tes1en-2	GAGGTAGTTCGCTACCTTAGCATAGAGCCCACCGCATCCC
Talen-tes1en-3	ACCAGTTACGCTAGGGATAAGGATGACGATGACAAGATGGC
Talen-tes1en-4	TAGCTATATTACCCTGTTATCATAGAGCCCACCGCATCCC
Tes1up-easy-1	atcttccagagattggatccgtTCGTAAGTCATGCCGTCCC
Tes1up-easy-2	taagagctcgatatctacgtatATAAACCAAGGCGAGCGT
Tes1dw-easy-1	TAGCTCGTCTTCACCTGCAGGCGAACTTACAGCCGCCAACG
Tes1dw-easy-2	ATTACTAGTCCGCGGGCATGCCTTAGAACCATCCCTCGGGT
